# Greenspaces and Health: Scoping Review of studies in Europe

**DOI:** 10.3389/phrs.2024.1606863

**Published:** 2024-05-20

**Authors:** Nicola Banwell, Sarah Michel, Nicolas Senn

**Affiliations:** ^1^ Interdisciplinary Centre for Research in Ethics (CIRE), University of Lausanne, Lausanne, Switzerland; ^2^ Department of Family Medicine, Center for Primary Care and Public Health (Unisanté), University of Lausanne, Lausanne, Switzerland

**Keywords:** greenspaces, contact with nature, health, behaviour change, co-benefits, blue spaces

## Abstract

**Objectives:**

Access to greenspaces and contact with nature can promote physical activity and have positive effects on physical and mental health. This scoping literature review aims to examine current evidence linking greenspaces and (a) behaviour change, (b) health outcomes and (c) co-benefits.

**Methods:**

This review was conducted in accordance with the PRISMA scoping review guidelines. Searches were conducted through PubMed and EMBASE databases for studies published between 2000 and March 2023 with a focus on Europe.

**Results:**

122 scientific articles and grey literature reports were identified. Access to greenspaces is positively associated with physical and mental health, and reduced risk of all-cause mortality and some non-communicable diseases. Greenspace quality is associated with increased physical activity and reduced risk of obesity. Nature-based therapies or green prescription are effective in improving mental health outcomes and overall health. Importantly, numerous co-benefits of greenspaces are identified.

**Conclusion:**

Increasing access to greenspaces for populations with particular attention to greenspace quality is important for co-benefits. Responsible governance and use of greenspaces are crucial to minimize public health risks and human disturbance of nature.

## Introduction

Contact with nature and greenspaces are important aspects of movement-friendly environments that promote behaviour change and positive physical and mental health [1–4]. The World Health Organization (WHO) recommends access to greenspace within 300 m of residences as a means to promote positive physical and mental health outcomes [5]. Identified health benefits of greenspaces include positive associations between exposure to urban greenspaces and physical activity, as well as protective effects for reducing negative health outcomes such as risks associated with mortality, mental health outcomes, stress, and cardiovascular diseases [1–3]. Furthermore, the WHO and scientists worldwide have highlighted the value of greenspaces for increasing biodiversity, as well as reducing air pollution and urban heat islands [6–8]. Such win-win interventions, here referred to as co-benefits, are both positive for human health and the environment [9]. Actors involved in urban planning and infrastructure management have important roles in ensuring the future development of greenspaces that simultaneously promote human health and wellbeing, and are environmentally sustainable [4]. However, a better understanding of the effectiveness of such strategies and the identification of specific interventions are necessary to inform the design of policies and programs that promote good physical and mental health while also protecting the environment. This scoping review summarises existing research on the effectiveness of movement-friendly environments, specifically contact with nature and greenspaces, in terms of impacts on behaviour change, health outcomes, and co-benefits in Europe.

## Methods

The focus of this scoping review was to understand the effectiveness of greenspaces from the perspective of movement-friendly infrastructure in relation to three specific outcomes are of interest, including: (a) impacts on behavioural change, (b) impact on physical and mental health, and (c) environmental co-benefits. Here greenspaces are understood as land openly accessible to the public that are designed to provide a natural environment for community members and access to spaces for recreation uses [8]. Examples of greenspaces include parks, gardens, public playgrounds, sports fields, hiking trails, etc. Urban greenspaces are considered as a sub-set of greenspaces which refers to vegetated land that surrounds or separates areas of concentrated residential or commercial activity [5, 8]. Blue space refers to visible surface waters in public space, this includes streams, lakes, rivers, waterfalls, etc., [10] and is included under the ‘greenspaces’ umbrella term. Finally, the quality of greenspaces refers to the density and diversity of biotic integrity (such as species richness and heterogeneity, and habitat heterogeneity), or pleasing aesthetic aspects of greenspaces, such as depth and lushness of greenery. Within this review, a prism of co-benefits has been adopted to support the identification of win-win interventions for human health and the environment. According to the Intergovernmental Panel on Climate Change (IPCC), co-benefits are “the positive effects that a policy or measure aimed at one objective might have on other objectives, thereby increasing the total benefits to the society or environment” ([11], p. 873). Co-benefits within the context of human health are interventions that are simultaneously beneficial for maintaining, restoring or improving both human health and the environment [9].

This review was conducted in tandem with a second scoping review focusing on mobility infrastructure (see Michel et al 2024 “Mobility Infrastructures and Health: Scoping Review of studies in Europe”). Both of these scoping reviews were carried out in parallel in accordance with the PRISMA guidelines for reporting scoping literature reviews [12]. Separate search strategies were developed for each review in collaboration with librarians specialized in health literature at Unisanté (University of Lausanne). For the review that is the focus of this article, combinations of key search terms such as “green space,” “green infrastructure,” “blue space,” “health behaviour change,” “physical health,” “mental health,” among others, were used to identify relevant literature (Supplementary Appendix S1). PubMed and EMBASE were the databases used for the search. The extraction (carried out on the 2nd March 2023), identification and analysis of relevant articles were conducted separately but in parallel for the two scoping reviews. Relevant articles were selected and classified according to the established criteria (Supplementary Appendix S2), including geographic focus on Europe and publication year of 2000 to March 2023. Even if this work corresponds methodologically to a scoping review, we adopted a rigorous literature search approach, comparable to systematic literature reviews, and used the Covidence® software to analyse and classify identified studies.

The initial search was complemented with a specific search dedicated to blue spaces to ensure an adequate number of studies related to blue spaces were identified. Literature included in relation to outcome (c) also drew on a pre-existing review conducted by members of the research team to complement the data [4, 13]. Grey literature from reputable international organisations in relevant domains (see Supplementary Appendix S2) were also used to complement the scientific literature. The strength of evidence was assessed qualitatively based on author consensus. The authors considered “strong evidence” to include systematic reviews and meta-analyses (particularly reviews of randomised control trials, nested case-control studies and prospective cohort studies).

## Results

**FIGURE 1:**
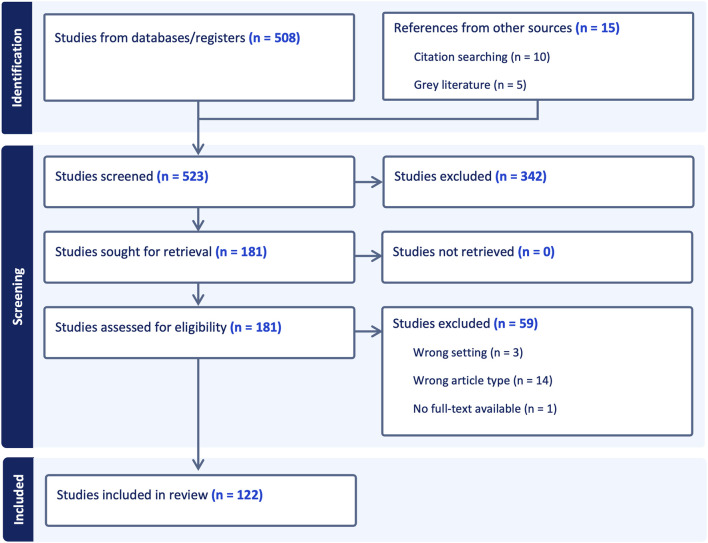
Flow diagram depicting the stages of study selection for the scoping review (Switzerland, 2024). A total of 523 abstracts were screened, with the initial search identifying 508 scientific studies and an additional 15 from other sources. Of the 181 studies retained for full text screening, 59 did not meet the established inclusion criteria, resulting in a total of 122 studies included in the review.

The scoping review identified a total of 122 combined scientific articles and grey literature reports (Figure 1). This included 107 scientific articles that examined the relationships between green and blue spaces and the three outcomes of interest. An additional 10 scientific articles and five grey literature reports were included through citation and grey literature searches. A total of 523 abstracts were screened, with the initial search identifying 508 scientific studies and an additional 15 from other sources. Of the 181 studies retained for full text screening, 59 did not meet the established inclusion criteria, resulting in a total of 122 studies included in the review.

Table 1 outlines the focus and types of articles identified in the review and Table 2 summarises the types of greenspace interventions identified. Results are presented with respect to the three outcomes of interest, including: (a) impacts on behavioural change, (b) impact on physical and mental health, and (c) environmental co-benefits.

**TABLE 1:** Focus and types of articles included in the review (Switzerland, 2024).

Features of identified scientific articles (Total = 117)	No of articles
Focus of article	
Greenspaces	92
Blue spaces	6
Both green and blue spaces	16
Intersection of green and blue spaces with active mobility infrastructures	8
Type of article	
*Literature reviews*	*67*
Meta-analysis	1
Systematic review and meta-analysis	8
Systematic review	30
Review of reviews	3
Scoping review	3
Rapid review	2
Narrative review	20
*Observational and experimental studies*	*50*
Case control studies	2
Cohort studies	15
Cross sectional studies	14
Non-randomised experimental studies	9
Randomized cross-over studies	2
Randomised controlled trials	4
Health impact assessments or modelling studies	4

**TABLE 2:** Greenspace interventions identified across articles included in the review (Switzerland, 2024).

Intervention type	Corresponding studies
Greenspace *Land openly accessible to the public that are designed to provide a natural environment for community members and access to spaces for recreation uses*	
Access or proximity to greenspace, greenness *Degree to which individuals, communities, or environments are connected to and surrounded by greenspaces and vegetation, common proxy measures include the NDVI*	(6,14–52)
Parks and community gardens *Park: public garden or area of land for recreation Community gardens: small plots of land integrated in urban neighborhoods managed collaboratively by residents*	(53–60)
Urban greenspace or infrastructure *Vegetated land that surrounds or separates areas of concentrated residential or commercial activity*	(5–8,10,18,21,56,61–72)
Density, diversity and quality of greenspace *Includes the density and diversity of biotic integrity (such as species richness and heterogeneity, and habitat heterogeneity), or pleasing aesthetic aspects of greenspaces, such as depth and lushness*	(7,8,51,61,62,68,73–84)
Playground Public space designed for engagement of children in play, recreation and physical activity	(14,32,85–87)
Blue space *Visible surface waters in public space, including streams, lakes, rivers, waterfalls, etc.*	
Access or exposure to blue space	(23,35,51,88–92)
Green and blue space	
Exposure to green and blue space	(10,20,21,32,35,62,88,93–96)
Nature-based interventions	
Physical activity and exercise interventions *Health interventions focused on promoting physical activity in greenspaces*	(97–110)
Walkability and cycling routes *Walkability: ease and convenience with which people can walk within a particular environment*	(111–113)
Nature-based therapy (green care) *Also known as green prescription or nature prescription which refers to a recommendation from a health or social professional for a patient to spend a fixed amount of time in a natural setting*	(22,97,114–123)
Contact with nature *Direct and intentional engagement or interaction between individuals and the natural environment*	(7,8,18,124–126)
Confounding factors	
Income and socioeconomic status	(7,8,24,61,113,127)
Gender	(8,128,129)
Ethnicity	(127,130)

### Behaviour Change

A small portion of academic and grey literature addressed behaviour change with regards to greenspaces and health (*n* = 8). Behaviors explored included first and foremost users and uses (such as the number of park visitors, types of activities and when greenspaces are used), as well as physical activity and the use of park and mobility infrastructure. With regards to behaviour change, public parks can serve as effective settings for interventions targeting youth to improve physical, mental, and emotional health outcomes [53]. Playground use and physical activity levels of youth in deprived neighbourhoods can be increased by promoting larger numbers of children to play through tailoring the design of playgrounds to include innovative designs, lendable sports material, and sports guidance in a safe environment [14, 85, 86]. Contrastingly, greenspaces can also be considered as unsafe spaces. For example, a survey of adolescents in high school settings in Scotland found that hanging around in a street or park more than once a week is associated with a threefold increase in intention to try e-cigarettes (OR 3.78; 99%CI = 1.93–7.39) [15]. Neighbourhood walkability is positively associated with higher numbers of park visitors and mean activity levels of visitors [88]. An Austrian study identified that cyclists tend to select routes with more green and aquatic areas, avoiding main roads and crossings including with fewer traffic lights and crossings [111]. They demonstrate a preference to use longer routes rather than the shortest possible ones when cycling infrastructure is present (e.g., bicycle lanes and pathways) and if these routes take them through green and flat areas [111]. Regeneration and upkeep of blue spaces are positively associated with changes toward healthier lifestyles and healthy urban environments [88]. Finally, contact with nature is suggested to improve sustainability-related behaviours [7], however further research is needed.

### Health Outcomes

Health outcomes, which were the main focus of the literature (*n* = 105), including: physical health, mental health, physical activity and obesity, mortality, and non-communicable diseases, respiratory and immune function, and childhood development and birth outcomes. These results are presented in relation to the type of intervention treated in the literature, including access or proximity to greenspace, urban greenspace, nature-based therapy, physical activity and exercise, blue space, and density and quality of greenspace.

#### Access or Proximity to Greenspace or Greenness

Access or proximity to greenspace appeared as the most studied intervention identified among the studies included in this scoping review. Often referred to as “greenness” exposure or contact with nature, commonly proxy measures include the Normalized Difference Vegetation Index (NDVI) [8]. Systematic reviews highlighted protective and positive relationships between greenness or contact with nature, and general overall health and wellbeing [6, 7, 16, 17, 124], as well as positive social benefits [7, 18]. Positive relationships are also found with exposure to combined blue and greenspaces [10, 61, 93]. However, it is important to note that some authors highlight that there is currently insufficient evidence to establish a longitudinal causal relationship between greenness and health [19, 20]. Table 3 provides a summary of the findings related to greenness and various health outcomes identified in this review.

**TABLE 3:** Summary of findings linking greenness and health outcomes identified in the review (Switzerland, 2024).

Outcome	Summary of findings	References
Mental Health	Improved mental health outcomes with increased exposure to greenspaces, biodiversity, and nature (elements for causal relationship established in adults but limited evidence in children)	(7,26,35,124)
	Improved affect with increased exposure to greenness	(94)
	Reduced risk of stress with increased exposure to nature and greenspaces	(7,18,124,125)
	Reduced risk of depression and anxiety with increased exposure to greenspaces in urban settings (including young adults)	(36,95)
	Reduced risk of psychosis with increased exposure to greenspaces in urban settings	(27)
Physical Activity	Increase of physical activity (frequency and intensity) with increased exposure to greenspaces (including forest, nature, community parks, sports fields, nature reserves, recreational parks, playgrounds, and school-based greenspaces)	(7,22,32,33,94)
	Mixed results for active transport (walking and cycling) and its association with greenspace access Obesity	(18)
	a. Limited evidence for a decrease of obesity associated to various greenness indexes b. Reduced prevalence of obesity with improved access to greenspaces among elderly c. Increased prevalence of obesity in higher risk of obesity persons among lower educated households with reduced access to greenspaces	a. (20,26,41–43,94) b. (41) c. (38)
All-Cause Mortality	Strong evidence for reduced risk of all-cause mortality with increased exposure to greenness	(8,16,28,29,47)
Non-Communicable Diseases	Reduced prevalence of atopic diseases, respiratory diseases, type two diabetes, and stroke associated to increased exposure to green and blue spaces in residential neighborhoods (strong evidence)	(10,28)
	Significant association with lower risk of cardiovascular disease	(20,28,30,31)
	Mixed results for cancer risks a. Potential increased risk of skin cancer associated with greenspace availability and accessibility b. Conflicting evidence of increased risk of lung cancer associated with greenspace availability and accessibility c. Conflicting or limited evidence for increased risk of breast and prostate cancer associated to outdoor activities	a. (46) b. (46). c. (10,28,46)
Youth Development and Pregnancy Outcomes	Improved youth development and decreased emotional and behavioural difficulties in children with higher NDVIs	(19,26)
	No association or mixed evidence for improved cognitive and brain development, academic achievement, absenteeism, social functioning, and cognitive skills with high exposure to greenspaces (mixed measures of exposure including NDVI, distance to greenspaces of varying types, and frequency of use)	(19,26)
	Increased healthy birth weight and reduced risk of small size with regards to gestational age with increased exposure to greenspaces and blue spaces	(16,48)
	Increased likelihood of breastfeeding with increased exposure to greenness	(49)
Life Satisfaction	Improved healthy development and life satisfaction (varies according to age) with increased access to greenspaces	(24)
Asthma and Allergies	Limited and conflicting evidence for increased asthma or allergy with increased exposure to greenness	(8,10,26,50,51)

#### Urban Greenspace and Urban Green Infrastructure

Strong evidence illustrates a positive association between urban greenspace and positive overall health [6, 8, 10, 62–65]. Protective effects have been found with regards to all-cause mortality [21, 66]. A quantitative estimate of European cities in 2015 found that meeting the WHO recommendation of access to green space could prevent 42,968 (95% CI 32296–64177) deaths annually (when measured using an NDVI proxy) [66]. This represents 2.3% (95% CI 1.7–3.4) of natural-cause mortality and 245 years of life lost per 100,000 inhabitants per year (95% CI 184–366) [66]. A quantitative estimate of all-cause mortality for adults aged 20 years or older in 93 European cities found that approximately 2,644 premature deaths (95% CI 2,444–2,824) due to urban heat islands could be prevented by increasing city tree coverage to 30% [21]. This corresponds to 1.84% (1.69–1.97) of all summer deaths and results in cooler city temperatures (mean 0.4°C; SD 0.2; range 0.0–1.3) [21]. Associations also exist with increased physical health [5, 7, 8] and positive mental health outcomes in adults and children [8, 63, 64]. For older adults, urban greenspaces are associated with higher physical activity, but mixed results have been found in terms of depression [63]. Larger urban parks, perceived quality of the urban greenspace, and presence of facilities such as walking trails, cycling routes, water areas, and playgrounds have been identified as positively related to higher levels of physical activity [8].

Community gardens are small plots of land integrated in urban neighbourhoods managed collaboratively by residents [54]. They offer a range of benefits to both individuals and communities, extending beyond physical health [4]. These include reduced depression, anxiety, and body mass index, and increased life satisfaction, quality of life, and sense of community [55]. Furthermore, they promote wellbeing through a sense of meaning, satisfaction, pride, social bonds and community involvement [131–134].

Urban greenspaces support child health and development [61, 63], and are important for social networks and inclusion for children and young people [8]. The use of anti-inflammatory sprays were higher in young children living in urban areas following high pollution days compared to those living near forest or national parks [67]. Urban greenspaces may also contribute to improved immune function [64].

#### Nature-Based Therapy and Green Care

There is a positive association between nature-based therapies and both physical health [22, 114] and mental health [97, 115–118]. Interventions identified in this review include “green prescription” or “nature prescription” (a recommendation from a health or social professional for a patient to spend a fixed amount of time in a natural setting), adventure-based activities, walking and relaxation in natural environments. A systematic review and meta-analysis of randomised control trials identified that green exercise and nature-based therapy are positively associated with reduced anxiety [Standard mean difference (SMD): 0.94; 95% CI: 0.94 to 0.01] and negative affect (SMD: 0.52; 95% CI: 0.77 to 0.26), as well as improving depressive mood (SMD: 0.64; 95% CI: 1.05 to 0.23) and positive affect (SMD: 0.95; 95% CI: 0.59–1.31) [97]. Strong and recent evidence in the form of a systematic review and meta-analysis illustrates that green prescription or nature prescription results in reduced anxiety and depression, reduced blood pressure, and increased daily step count of an average of 900 steps [118]. This meta-analysis identified a moderate reduction of depression with a SMD of −0.50 (ranging from −0.84 to −0.16) post-intervention. Similarly, moderate to large reductions in anxiety scores were identified post-intervention with a standardised mean difference of −0.57 (ranging from −1.12 to −0.03) [118]. For patients with well-defined diseases, nature-based therapy has been found to aid with decreasing psychiatric symptoms, anger, substance abuse (including craving and relapse), and improve outcomes for abstinence from drugs, mood and anxiety disorders, behavioural and personality disorders, acquired brain injury and youth delinquency [115]. The findings of these reviews are supported by several non-randomised experimental studies which have identified the value of nature-based therapy for reducing stress and improving psychological health [116, 117].

#### Physical Activity and Exercise Interventions

A positive association exists between outdoor activities in natural environments and overall health [22]. A systematic review of randomised control trials and quasi-experimental studies of exercise interventions found an overall indication of improvement in wellbeing, mood, and physical performance [98]. The design and size of playgrounds [14, 85, 86] and the presence of active children [14] have been found to be important factors for physical activity. Innovative playground design is associated with moderate and vigorous activity levels, playground size is strongly linked to the number of visitors, and designing playgrounds for adults to include spaces for adults and caregivers to sit or gather is as important as designing for children to increase visiting hours [86].

Moderate and vigorous physical activity has been found to more likely take place in parks and fields compared to streets and other urban spaces [99, 100]. A study in the United Kingdom found that walking in an urban street compared to an urban park can result in higher respiratory symptoms among sufferers of chronic obstructive pulmonary disease [101]. Walking in an urban park was associated with improved respiratory function, however, these benefits were diminished when this was followed by a walk on an urban street [101]. A randomised control trial in Lithuania found that physical activity in a green environment with lower noise and air pollution is associated with a more positive impact on the stress and hemodynamic parameters of patients with coronary artery disease when compared to physical activities in urban environments [102]. A randomised control trial comparing lunchtime walks in nature compared to the built environment found that perceived mental health was improved for the nature walking group only [103]. Sitting or walking in nature for 10 min, compared to urban environments, was found to improve mental wellbeing in college students [104]. Additionally, a randomized cross-over study in Spain found that short walks in blue spaces is associated with a positive impact on both mood and wellbeing [105]. This finding is supported by systematic reviews [97, 98].

#### Access or Exposure to Blue Space

Some studies have demonstrated that there is evidence of associations between blue spaces and overall health [89, 90]. Others highlight the heterogeneity of evidence which makes it difficult to draw clear conclusions. Despite this the balance of evidence suggests a positive association between health and blue spaces [91]. All studies examining mental health suggest a benefit for overall mental health, and reduced stress and mood disturbance [23, 91, 92]. Other associations identified include a positive association with physical activity [91]. Insufficient evidence exists to draw conclusions regarding benefits for diabetes and cardiovascular disease [91].

#### Density and Quality of Greenspace

The evidence surrounding the density and quality of greenspace indicates that there is an overall positive association with multiple physical and mental health outcomes [7, 61, 68]. The perceived quality of greenspace, particularly biotic integrity (such as species richness and heterogeneity, and habitat heterogeneity), and pleasing aesthetic aspects of greenspaces (such as depth and lushness of greenery in parks), have been identified as important for self-reported health, reduced psychological distress and encouragement of physical activity. Increased physical activity and reduced risk of obesity has been found as being related to the quality of greenspace activity [7]. This is also demonstrated by a cross-sectional study of cities in Europe. Across the eight European cities studied, it was identified that individuals living in areas with high greenness have 3.3 times the odds of engaging in physical activity compared to those living in areas with low greenness (OR: 3.32; 2.46 to 4.50; *p* < 0.001) [73]. Furthermore, individuals living in areas with high greenness have 0.63 times the odds of obesity compared to those living in areas with low greenness [73]. This scoping review highlighted an important limitation with regards to proxy measures for greenness. In particular, two greenspaces with the same NVDI or percentage of canopy coverage may in reality reflect very different levels of quality, particularly in relation to biodiversity, and vegetation and fauna compositions. Relying on these proxy measures presents major limitations in terms of the comparability of the diversity of greenspaces, their classification and their quality (particularly biodiversity).

#### Differing Health Benefits in Specific Populations

Greenspaces bring significantly greater benefits for particular groups. Contact with nature has a stronger protective effect on the physical health of individuals from low socioeconomic backgrounds and minority groups [7, 8, 127]. In European cities communities with lower socio-economic status generally have fewer and lower quality greenspaces [61]. A large cross-sectional study in Basel, Switzerland found that the association between residential greenness and life satisfaction varies based on age group, household income, and financial concerns [24]. Within this context, residential greenness was positively associated with life satisfaction among those with high household income and fewer financial worries. A negative association was found between life satisfaction and residential greenness for those between 18 and 29 years of age and those with more financial concerns [24]. Specifically, living closer to a forest as opposed to a park or agricultural area, was associated with lower life satisfaction in the young adults aged 18–29 years. According to the authors of this study, this indicates differing perceptions of greenspace between younger and older people, and that perhaps younger people living further from city centres in greener areas feel more isolated. This suggests that access to greenspaces, particularly parks, are an important feature for healthy development and life satisfaction, however this may differ with age.

Having good access to greenspaces helps to reduce the negative impact of socioeconomic inequality on mental wellbeing by 40% [25]. Several studies have found that proximity to natural environments has a stronger association with health outcomes among groups with lower income or education, and that loss of biodiversity may disproportionately impact the health and wellbeing of the poorest [7]. Access to greenspaces is associated with improved mental health in women [8]. Furthermore, studies in Europe have found positive associations between access to nearby green space and reduced blood pressure and depression in pregnant women, particularly among disadvantaged groups [8]. Ethnic groups have been found to have lower levels of cycling and gardening compared to non-minority groups [130]. Finally, walking generally increases with perceived safety for women and having a park within walking distance for men [128].

### Co-Benefits

Environmental co-benefits and ecosystem services appeared in 22 scientific articles and grey literature reports. Topics covered included biodiversity and immune function, climate change mitigation and adaptation, and ecosystem services (air pollution, heat and noise, and water run-off regulation). Trade-offs for the health of ecosystems and humans were also identified including vector borne disease, increased presence of allergens and risk of UV exposure, and the negative impact of human activity on natural spaces and species.

Greenspaces provide ecosystem services that are important for human health and wellbeing. They can serve important functions for air quality regulation though the pathways are complex, and the results are somewhat mixed [18, 62, 89]. Trees and other plants can mitigate air pollution by absorbing gases and particulate matter [18, 62]. However, they can also contribute to air pollution by releasing hydrocarbons and pollen [18]. It is well established that green and blue spaces have important roles in reducing the urban heat island affect and heat stress in general [6, 56, 62, 69, 89, 94]. They also help to regulate water-runoff [6, 62] and attenuate noise (excluding deciduous vegetation) [62, 74, 94].

Greenspaces and urban green infrastructure are important climate change mitigation and adaptation strategies. They support adaptation to various climate hazards such as flooding, drought, heat and precipitation variability and can be implemented by national and local governments as a key response to climate change [6]. Urban vegetation can contribute indirectly to climate change mitigation and improved air quality by providing passive cooling and thus reducing building energy demand [18]. As climate change continues to progress, loss of access to greenspace due to storm damage, drought, and wildfires is likely to increase, which will have negative impacts for human and ecosystem health [6].

Urban green infrastructure and blue space regeneration can enhance ecological health and biodiversity [17, 68, 88, 90]. Biodiversity can contribute to human health in a variety of ways, such as through cultural and spiritual values, social connectedness, and immune resilience and functioning [7, 13, 75–78]. It is important to note that the direction of the association between ecosystem health and human health can depend on the state of health of ecosystems, in general positive ecosystem health was found to be linked to positive human health and negative ecosystem health was linked to negative human health.

Green and blue spaces also present potential public health risks such as increased risks for exposure to disease vectors (e.g., tics) and zoonotic diseases, allergens, algae, excessive UV exposure, and drowning [6, 8, 64]. Several studies in this review highlighted activity of disease vectors in parks and urban greenspaces [79, 80] including increased activity associated with climate change [6, 81]. Additionally, increased or expanded use of greenspaces may present important trade-offs and negative impacts for animal species and their habitats. More specifically, nature-based recreation such as wildlife viewing, hiking, running, cycling, canoeing, horse riding, dog walking, and free-ride snow sports have been found to negatively impact bird populations, some of which are threatened species [106, 107].

## Discussion

**FIGURE 2:**
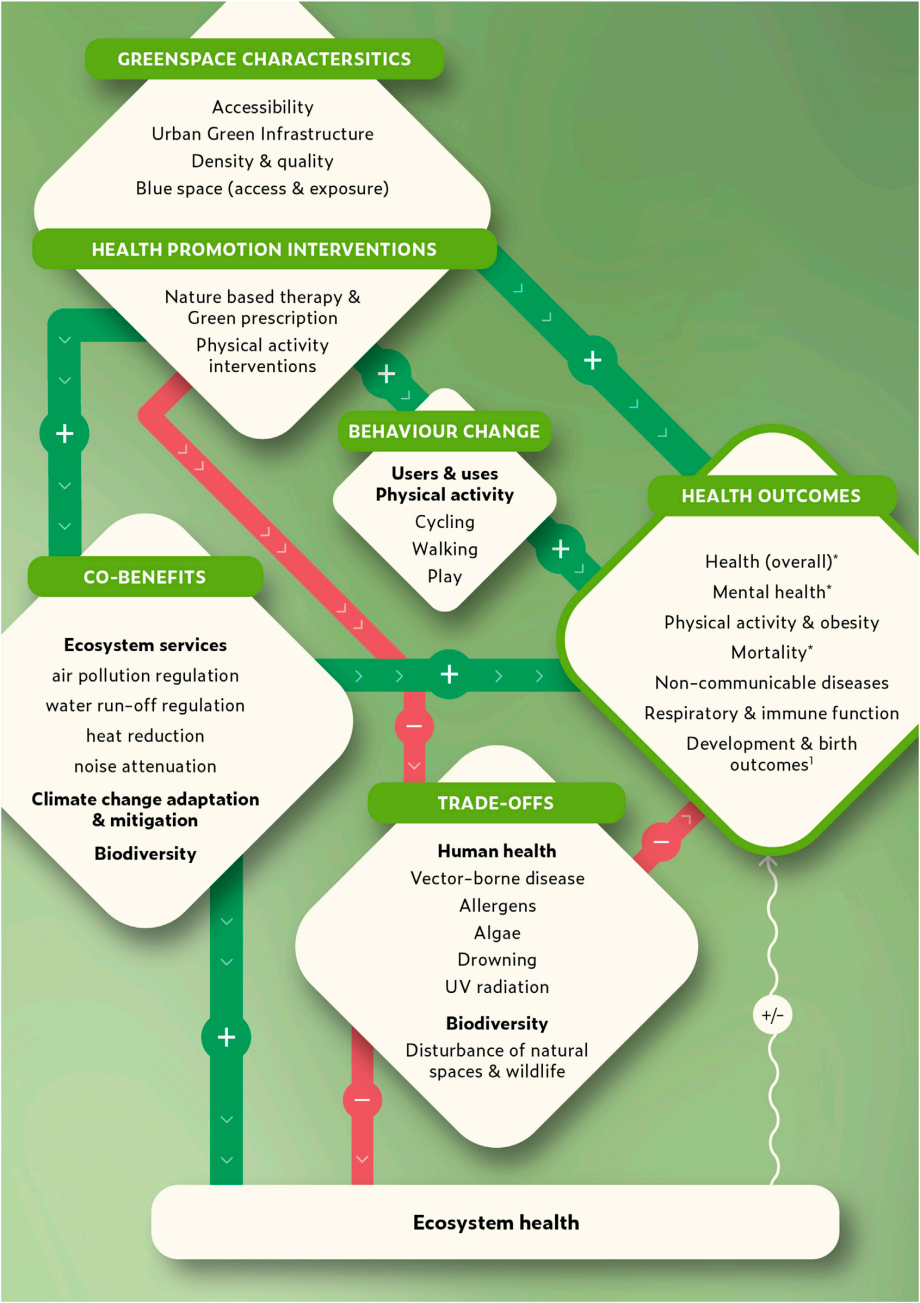
Associations between greenspaces, behaviour change, health outcomes and co-benefits (Switzerland, 2024). Green arrows indicate positive associations, red indicates negative association and the white arrow indicates mixed associations. In the case of the association between ecosystem health and human health can depend on the state of health of ecosystems, in general positive ecosystem health was found to be linked to positive human health and negative ecosystem health was linked to negative human health. *Strong evidence in the form of meta-analyses exists for the relationship between greenspace and overall health, mental health and all-cause mortality. ^1^No literature pertaining to blue space and development and birth outcomes was identified.

This scoping review aimed to synthesize existing research relating to the effectiveness of movement-friendly environments, specifically greenspaces and contact with nature, in terms of their impact on behaviour change, health outcomes and co-benefits in Europe (summarized in Figure 2). A prism of co-benefits was adopted to identify win-win intervention strategies for human health and the environment.

Regarding greenspaces and health behavior change, public parks can be effective settings for interventions targeting youth to improve physical and mental health [53]. Tailoring the design of playgrounds and promoting larger numbers of children to play can increase playground use and physical activity levels of youth in deprived neighborhoods [14, 85, 86]. Neighbourhood walkability [88], contact with nature [7] and the regeneration and upkeep of blue spaces [88] are positively associated with healthier or more sustainable lifestyles, and healthy urban environments.

Access and exposure to greenspaces has been identified as having positive relationships with health and wellbeing [6, 7, 16–18, 124], mental health [7, 18, 26, 27, 94, 95, 124, 125], reduced all-cause mortality [8, 16, 28, 29], and protective effects against certain non-communicable diseases [20, 28, 30, 31]. Greenspaces are also positively associated with physical activity [7, 14, 18, 22, 32–34, 94] and have a mixed relationship with obesity, with some studies suggesting lack of access to greenspaces as a mediating factor for higher risk of obesity in households with lower education. Nature-based therapies, green care, or green prescription are effective “activators” for improving mental health outcomes and overall health [22, 97, 114, 118]. The balance of evidence suggests that access or exposure to blue spaces has positive associations with overall health benefits, but more research is needed to draw definitive conclusions about its potential benefits for diabetes and cardiovascular disease [23, 91, 92]. The density and quality of greenspace is associated with multiple physical and mental health outcomes, with lower-income or less-educated groups benefiting the most [7, 8, 61, 68, 128].

Greenspaces provide essential ecosystem services that are important for human health and wellbeing, such as air quality regulation [18, 62, 89], heat reduction [6, 56, 62, 69, 89, 94], noise attenuation [62, 74, 94], and water runoff regulation [6, 62]. They are important strategies for climate change mitigation and adaptation [6] and create co-benefits such as enhancing ecological health and biodiversity [17, 68, 88, 90]. However, they can also contribute to public health risks through exposure to vector-borne disease, allergens, and UV radiation [6, 8, 64, 79–81]. Responsible governance, management and developing biodiverse greenspaces is imperative to minimize the impact of human disturbance through the use of greenspaces, which poses a serious threat to wildlife [106, 107].

Based on these findings, it is important to increase access to greenspaces for populations, particularly those from low socioeconomic backgrounds and minority groups, paying attention to the quality of greenspaces, in terms of both density and diversity, particularly in deprived neighbourhoods. Nature-based therapy and green prescription, exercise interventions, and innovative playground design can also encourage outdoor activities and behaviour change. The context and specific needs of different groups of users should be considered when planning greenspace interventions. Additionally, it is important to note a major limitation exists regarding the comparability of the diversity of greenspaces, how they are classified, and their quality when common proxy measures for greenness are used. While several studies were identified highlighting trade-offs in the form of public health risks, limited research was found linking health promoting activities in greenspaces to human disturbance of species and nature. However, the few studies identified indicate that this is an important factor to be considered in the governance, management, and use of greenspaces.

Further research is necessary to aid public health decision-makers to distinguish between types of urban green infrastructure (e.g., street trees, parks, playgrounds, etc.,) and their effectiveness and health benefits. There is limited research linking greenspaces, sustainable and pro-environmental behaviours and health outcomes. Furthermore, the proxy measures often employed in the identified studies (such as NDVI, percentage of canopy coverage, etc.,) present major limitations in terms of the comparability of the diversity of greenspaces, how they are classified and their quality (particularly their biodiversity). For example, two spaces with the same NVDI or percentage of canopy coverage may in reality reflect very different levels of biodiversity, vegetation and fauna compositions, and different health outcomes. Finally, the restriction of the search criteria to studies relating to European countries presents potential limitations regarding the inclusion or exclusion of some literature, as well as the transferability of some of the results presented in this review to global contexts. The authors sought to address this challenge by drawing on key sources of international literature from the WHO and IPCC. On the other hand, it provides data to policymakers that might better reflect their local (European) contexts.

Access to greenspaces has been found to have positive associations with overall health and wellbeing, mental health outcomes, and physical health, including a reduced risk of all-cause mortality and numerous non-communicable diseases. They provide ecosystem services and co-benefits that are important for human and ecosystem health. Finally, it is important to note the public health risks of greenspaces and the potential for human activity to disturb natural environments. Responsible governance, management, and use of greenspaces is crucial to minimize the impact of human disturbance on wildlife and to maximize the benefits to human health and wellbeing.
