# Supplementary material for: Greenspaces and Health: Scoping Review of studies in Europe
Source: Public Health Rev. 2024 May 20;45:1606863. doi: 10.3389/phrs.2024.1606863 (PMC11144923; doi:10.3389/phrs.2024.1606863)
Supplement: Supplementary file 1 [file DataSheet1.docx]

**SUPPLEMENTARY MATERIAL:**

**Greenspaces and Health: Scoping Review of studies in Europe**

Banwell, Nicola – Interdisciplinary Centre for Research in Ethics (CIRE), University of Lausanne, Lausanne, Switzerland; nicola.banwell@unil.ch

Michel, Sarah - Department of Family Medicine, Center for Primary Care and Public Health (Unisanté), University of Lausanne, Lausanne, Switzerland; sarah.michel@unil.ch

Senn, Nicolas – Department of Family Medicine, Center for Primary Care and Public Health (Unisanté), University of Lausanne, Lausanne, Switzerland; nicolas.senn@unisante.ch

**Appendix S1 – Search Strategy**

**Key search terms**

| **Topic** | **Key search terms** |
| --- | --- |
| *Review focus* | |
| 2. Green and blue spaces | Green space, greenspace, greening, greenness, parks, nature area, urban nature, urban garden, community garden, green infrastructure, biodiversity, forest, national park, field, one health, urban agriculture, community garden, urban farming, garden, permaculture, vegetation, landscape, built environment, bluespace, blue space, blue infrastructure |
| *Outcome:* | |
| a) impacts on behavioural change | Mobility behaviour change, Health behaviour change, pro-environmental behaviour, health promoting behaviour, healthy behaviour, physical activity |
| b) impact on physical and mental health | Physical health, cardiovascular disease, type 2 diabetes, obesity, cancer, mental health, anxiety, depression, stress, hypertension, well-being, wellness, trauma, injury, pulmonary diseases, asthma, allergies (immune diseases) |
| c) environmental co-benefits relating to biodiversity and climate change | co-benefits, cobenefits, biodiversity, human nature contact, nature, climate change, greenhouse gas emissions, air pollution, soil pollution |

**An example of the search syntax used for PubMed is shown below.**

("Biodiversity"[Majr:NoExp] OR "Built Environment"[Mesh:NoExp] OR “Forests”[Mesh] OR “Gardening”[Mesh] OR “Gardens”[Mesh] OR “Nature”[Majr] OR "Parks, Recreational"[Majr] OR "green space*"[tiab] OR “greenspace*”[tiab] OR “green area*”[tiab] OR “green infrastructure*”[tiab] OR "garden*"[ti] OR “permaculture”[tiab] OR “Trees”[Mesh] OR "biodivers*"[ti] OR "park"[tiab] OR "parkland"[tiab] OR "parks"[tiab] OR “blue infrastructure*”[tiab] OR “blue space*”[tiab] OR “bluespace*”[tiab] OR “greenery”[tiab] OR “greening”[tiab] OR “greenness”[tiab] OR “open space*”[tiab] OR "forest*"[ti] OR "urban nature"[tiab] OR "natural environment"[tiab] OR "nature"[ti])

AND

(“Health behavior”[Mesh] OR ”health-related behavio*”[ti] OR “healthy behavio*”[ti] OR “behavior change*”[ti] OR “behaviour change*”[ti] OR “environmental awareness”[ti] OR “health behavio*”[ti] OR “health promoting behavio*”[ti] OR “mobility behaviour change”[ti] OR “multiple behavio*”[ti] OR “multiple health behavio*”[ti] OR “pro-environmental behavio*”[ti] OR “proenvironmental behavio*”[ti] OR “Leisure Activities”[Mesh:NoExp] OR “leisure activit*”[ti] OR "Sports"[Mesh:NoExp] OR “sport*”[tiab] OR “running”[tiab] OR “walk”[tiab] OR “walking”[tiab] OR “smoking”[tiab] OR "social connect*"[tiab] OR "Social Cohesion"[Mesh] OR "social cohesion"[tiab] OR “Social skills”[Mesh] OR “social skills”[tiab] OR “prosocial behavio*”[tiab] OR “active lifestyle”[tiab] OR “Sleep”[Mesh] OR “sleep”[tiab] OR “Exercise”[Mesh] OR “exercise*”[ti] OR “physical activit*”[tiab] OR “Diet”[Mesh:NoExp] OR “diet”[tiab] OR “eating”[tiab] OR “nutrition”[tiab] OR "Feeding Behavior"[Mesh])

AND

("Health"[MeSH] OR "Health Status"[MeSH:NoExp] OR “health” [ti] OR "Chronic Disease"[MeSH] OR "Disease"[MeSH] OR "disease*"[ti] OR "Mental Disorders"[MeSH] OR "mental well-being"[tiab] OR "mental wellbeing"[tiab] OR “mental illness”[tiab] OR "psychiatric disorders"[tiab] OR "anxiet*"[tiab] OR "depress*"[tiab] OR "mood*"[tiab] OR “psychological wellbeing”[tiab] OR “psychological well-being”[tiab] OR “emotional well-being”[tiab] OR “emotional wellbeing”[tiab] OR "morbidity"[tiab] OR "mortality"[tiab] OR "Morbidity"[MeSH:noexp] OR "Mortality"[MeSH:noexp] OR "birth outcome*"[tiab] “pregnancy”[tiab] OR "Pregnancy Outcome"[Mesh:NoExp] OR "allerg*"[tiab] OR "rhinitis, allergic, seasonal"[MeSH] OR "Allergy and Immunology"[MeSH:NoExp] OR "asthma*"[tiab] OR "Body Mass Index"[MeSH] OR "Body Mass Index"[tiab] OR "Obesity"[Majr] OR "obesi*"[tiab] OR "Quality of life"[MeSH Terms] OR "quality of life"[tiab] OR "cancer"[ti] OR "diabet*"[ti] OR "cardiovascular"[tiab] OR "Blood Pressure"[tiab] OR "Blood Pressure"[MeSH:NoExp])

AND

("Europe"[Mesh] OR Europe*[tiab] OR Andorra[tiab] OR Austria*[tiab] OR Balkan[tiab] OR Belgium[tiab] OR Britain[tiab] OR Danish[tiab] OR Denmark[tiab] OR England[tiab] OR Finland[tiab] OR France[tiab] OR French[tiab] OR German*[tiab] OR Gibraltar[tiab] OR "United Kingdom"[tiab] OR Greece[tiab] OR Iceland[tiab] OR Ireland[tiab] OR Italy[tiab] OR Liechtenstein[tiab] OR Luxembourg[tiab] OR "Mediterranean Region"[tiab] OR Monaco[tiab] OR Netherlands[tiab] OR "Nordic Countries"[tiab] OR Norway[tiab] OR Portug*[tiab] OR "San Marino"[tiab] OR Scandinavia* OR Spain[tiab] OR Spanish[tiab] OR Sweden OR Swiss[tiab] OR Switzerland[tiab] OR Transcaucasia[tiab] OR Vatican[tiab])

**Appendix S2 – Inclusion and exclusion criteria**

| **Inclusion criteria** | **Exclusion criteria** |
| --- | --- |
| Peer-reviewed academic articles including:   - Research studies with various design, interventional and observational (e.g. randomised control trials, case-control studies, pre-post studies, observational studies) - Modelling studies linking to health and at least one of the two focus areas - Literature reviews articles of all types | Expert commentaries and non-empirical academic articles, case reports |
| Grey literature from reputable international organisations in relevant domains including: WHO, IPCC, IUCN, UN Biodiv, IPBES, UN Habitat, UNEP, European Environment Agency. | Modelling studies not linking to health |
| Policy recommendations and guidelines when available | Non-peer reviewed publications including conference proceedings. |
| Published since 2000 | Articles not written in French and English |
| Swtizerland (in particular for illustrative case studies and examples) and Europe |  |
| Focus of the literature is on behavioural change, health impacts and environmental co-benefits of mobility infrastructure and/or greenspaces and parks |  |
